# Perceived Patient Workload and Its Impact on Outcomes During New Cancer Patient Visits: Analysis of a Convenience Sample

**DOI:** 10.2196/49490

**Published:** 2023-08-18

**Authors:** Safa Elkefi, Onur Asan

**Affiliations:** 1 School of Systems and Enterprises Stevens Institute of Technology Hoboken, NJ United States

**Keywords:** health care, cancer patients’ workload, trust, satisfaction, health information technology

## Abstract

**Background:**

Studies exploring the workload in health care focus on the doctors’ perspectives. The ecology of the health care environment is critical and different for doctors and patients.

**Objective:**

In this study, we explore the patient workload among newly diagnosed patients with cancer during their first visit and its impact on the patient’s perceptions of the quality of care (their trust in their doctors, their satisfaction with the care visits, their perception of technology use).

**Methods:**

We collected data from the Hackensack Meridian Health, John Theurer Cancer Center between February 2021 and May 2022. The technology use considered during the visit is related to doctors’ use of electronic health records. A total of 135 participants were included in the study. Most participants were 50-64 years old (n=91, 67.41%). A majority (n=81, 60%) of them were White, and only (n=16, 11.85%) went to graduate schools.

**Results:**

The findings captured the significant effect of overall workload on trust in doctors and perception of health IT use within the visits. On the other hand, the overall workload did not impact patients’ satisfaction during the visit. A total of 80% (n=108) of patients experienced an overall high level of workload. Despite almost 55% (n=75) of them experiencing a high mental load, 71.1% (n=96) reported low levels of effort, 89% (n=120) experienced low time pressure, 85.2% (n=115) experienced low frustration levels, and 69.6% (n=94) experienced low physical activity. The more overall workload patients felt, the less they trusted their doctors (odds ratio [OR] 0.059, 95% CI 0.001-2.34; *P*=.007). Low trust was also associated with the demanding mental tasks in the visits (OR 0.055, 95% CI 0.002-2.64; *P*<.001), the physical load (OR 0.194, 95% CI 0.004-4.23; *P*<.001), the time load (OR 0.183, 95% CI 0.02-2.35; *P=*.046) the effort needed to cope with the environment (OR 0.163, 95% CI 0.05-1.69; *P*<.001), and the frustration levels (OR 0.323, 95% CI 0.04-2.55; *P*=.03). The patients’ perceptions of electronic health record use during the visit were negatively impacted by the overall workload experienced by the patients (OR 0.315, 95% CI 0.08-6.35; *P*=.01) and the high frustration level experienced (OR 0.111, 95% CI 0.015-3.75; *P*<.001).

**Conclusions:**

The study’s findings established pathways for future research and have implications for cancer patients’ workload. Better technology design and use can minimize perceived workload, which might contribute to the trust relationship between doctors and patients in this critical environment. Future human factors work needs to explore the workload and driving factors in longitudinal studies and assess whether these workloads might contribute to unintended patient outcomes and medical errors.

## Introduction

### Background

Cancer is a major global public health issue in modern medicine [1]. Based on a report by the National Cancer Institute, 18.1 million new cancer cases were recorded in 2018, with 9.5 million cancer-related deaths worldwide [2]. This number is expected to rise to more than 20 million new cancer cases by 2025 [3] and 29.5 million by 2040 [2]. After initial diagnosis, clinical information becomes complex, leading to increasingly complicated treatment recommendations for patients with cancer [4]. The ecology of the first visits after diagnosis is unique [5] since patients experience significant life disruptions [6]. These disruptions can come from disease symptoms and the burden of treatment-related decision-making [6]. In these new cases, a diagnosis threatens their physical well-being and their sense of cognitive and emotional well-being [1]. In addition, they have difficulty understanding the medical information and generally report dissatisfaction with the delays in prognosis and follow-ups [7]. This results in psychosocial concerns among patients [1,8]; they experience high distress, emotional stress, uncertainty about mortality, and a disturbing social life [9,10]. These cognitive and emotional workloads might overburden patients with cancer, resulting in a higher likelihood of nonadherence to treatment plans [11].

Within the context of cancer care, the link between people, work, and goals is complex and multidimensional. Studying how humans interact with their environment, including the tools, technology, and systems they use, is referred to as human factors. Human factors are critical in understanding the interactions between health care personnel, patients, and the broader health care system in cancer care [12]. For example, according to human factors research, effective communication and teamwork among health care workers are critical for obtaining optimal patient outcomes in cancer care settings [12]. Furthermore, creating clear goals and addressing cancer patient needs and preferences during the visit is critical for increasing patient engagement and outcomes. Human factors study aids in the identification of potential hurdles and challenges in the cancer care process, such as workload, information overload, and other issues [12]. By addressing these issues, health care institutions can increase patient safety, reduce medical errors, and improve overall cancer treatment quality [12].

Cancer visits involve 3 main parties: doctors delivering information, patients, and families receiving the services under emotionally pressured situations, and technology supporting the information delivery and overall care. The primary interaction occurs between the doctor and patient, discussing the new diagnosis and future treatment plan. Electronic health records (EHRs) are the main technologies used by doctors during the visit. However, some studies reported that EHR use might increase doctors’ cognitive workload [13], negatively impact doctor-patient communication [14], and create less attentive doctors during the visit. Studies also showed that oncologist doctors use EHRs less than primary care doctors during these emotional visits to avoid the aforementioned negative aspects [15].

To deliver optimal holistic cancer care, it remains essential to take actions centered around the patients, mirroring their needs and expectations [16]. Patient-centered care is based on respect for patients’ expectations and values. It aims to provide them with the needed education and information, ensure their continuous secure access to care, and involve their families to support their emotional well-being [17]. In cancer care, the relationship between doctors and patients discriminates between 2 underlying dimensions: technical, related to the medical situation, and affective, pertaining to the relations and emotions of the patients [18]. Thus, the rational-consumer patient-centered care model would not suit oncology settings [19]. Patient-centered care has proved to be important in improving health care outcomes. When doctors engage in effective communication and shared decision-making and demonstrate trust in their patients, patients show more efficacy in self-management and have better psychological and physical health outcomes [20-23]. Patient-centered care should also be studied from a patient ergonomics perspective. Patient ergonomics is the application of human factors or related disciplines to study and improve patients’ and other nonprofessionals’ performance of effortful work activities in pursuit of health goals [24,25]. A central emerging concept of societal views of health care considers that the patients actively perform “work” to achieve health-related goals and objectives [26]. By that, human factors position the patients in the center of the work system aiming to improve their experience with the load of work assigned [24,27]. In highly sensitive situations like cancer care, this paradigm can help us better understand the dynamics between the 3 actors of the visits (doctor, patient, technology) and how their interaction can influence critical outcomes like quality of care, trust of doctors, and acceptability or perception of technology use.

Advancements in digital communication and medical technologies have led to digitalizing health care [14,28]. With the increased adoption and use rate of EHRs in cancer care, oncologists can use the provided data in the critical decision-making process and support their workload [29]. In a study by Mazur et al [30], the enhancement of EHR systems’ usability was associated with better oncologist doctors’ cognitive workload and performance. Studies also explore how EHR influences doctors’ cognitive workload and performance in various settings [31]. However, no study has explored patients’ overall workload as well as how technology use impacts their workload during the visits. Given the importance of supporting new cancer patients’ “work” success, a holistic approach that recognizes the impact of workload on care outcomes in the first visits remains important. Therefore, this cross-sectional survey-based study investigates the workload of cancer patients in new cancer patient visits and its association with the following outcomes: trust in care doctors, satisfaction with the care delivered, and their perception of the technology (EHR) used in cancer care.

### Theory and Hypotheses

It is critical to understand the users’ workload while performing a task using technology, especially in highly complex environments such as health care. The purpose of a workload evaluation is to determine the user’s workload while he or she is working on a given task using or utilizing a system or technology [32]. The concept of workload has been described as “the cost of performing a task in this way that reduces the capacity to perform other tasks that use the same processing resource” [32]. The workload is measured to assess the performance of users and systems [33]. Since working memory is limited, distractions, new information, and complex information can interfere with clinical decision-making and can result in errors [34]. Cognitive load is a measure of how many cognitive resources are used during thinking, learning, problem-solving, and reasoning [35]. Studies used subjective workload assessments such as NASA TLX (National Aeronautics and Space Administration Task Load Index) in various contexts, including aviation and health care [36,37]. In health care, most studies focus on measuring clinician workload [38]. However, there is a lack of studies focusing on understanding patients’ perspectives of workload. Especially no study measured patients’ workload in high-anxiety environments such as cancer care [39].

Problems related to workload-related vulnerabilities are discussed in cancer care literature [40]. Discovering a cancer diagnosis brings emotional pressure to new patients and causes a stress load that makes them experience difficulty finding their emotional stability [41]. In addition, trust in doctors is an important component of patient-centered care as it plays a pivotal role in the success of cancer treatment strategies [42]. In this study, we hypothesize that high levels of workload during the initial visit would negatively impact newly diagnosed cancer patients’ trust in their doctors on the first visit after diagnosis (hypothesis 1).

Furthermore, as a new cancer diagnosis is disorienting for patients, newly diagnosed patients might experience high levels of anxiety and depression [43]. With the triggered unmet physical, psychological, and informational needs, patients require much more attention than what they receive [44]. In addition, new patients report dissatisfaction with care systems (delays in diagnosis, follow-ups, etc) driven by confusing, unclear processes and inefficient procedures [7,45]. We hypothesize that satisfaction with the care visit is negatively impacted by the workload experiences of newly diagnosed cancer patients in the very first visits after diagnosis (hypothesis 2).

Finally, we showed in a previous review that health information technology is used in cancer care to propose solutions that can strengthen the cancer patients’ relationship with their doctors, empower their well-being and build a structured target-oriented care process for them [46]. Despite its benefits, using EHR extensively during these highly emotional visits might have negative consequences. Newly diagnosed cancer patients’ experienced physical, mental, and emotional pressure can affect their perceptions towards using technologies like EHRs during the visits. Thus, we hypothesize that newly diagnosed cancer patients’ high workload negatively impacts their perception of EHR use during the very first visits after diagnosis (hypothesis 3).

**Figure 1:**
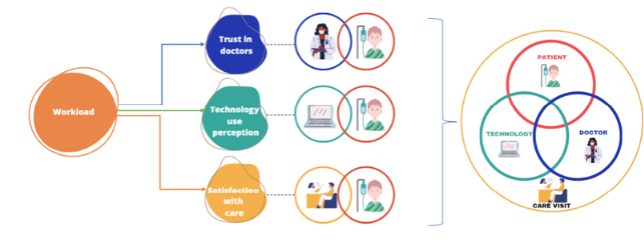
Overall conceptual framework of the study.

To sum up, the 3 hypotheses of this study investigate the interrelation between the 3 actors of the visit: new cancer patients, doctors, and technology. Figure 1 details the conceptual framework followed.

## Methods

This study took place at the Hackensack Meridian Health, John Theurer Cancer Center. The setup of the patients’ rooms in the cancer center is standard and identical to each other with an EHR system in the room.

### Ethics Approval

The study obtained ethical approval from both the Stevens Institute of Technology and the Hackensack Meridian John Theurer Cancer Center IRB offices (IRB ID 00011536).

### Data Collection and Participants

This study is part of a federal grant that was funded in May 2020. Due to COVID-19 restrictions, the data collection started with 7 months delay in February 2021. We used a convenience sampling method to recruit newly diagnosed patients when they came for their very first visit with a cancer doctor at the Cancer Center. Convenience sampling is a nonprobability sampling technique that involves selecting your research sample based on convenience and accessibility [45]. The inclusion and exclusion criteria included (1) having the new cancer patient visit, (2) understanding English, (3) being between 18-65 years old, and (4) not having any dementia and cognitive impairments. Patients who have upcoming visits are first contacted by phone and informed about the study. If they agreed, they completed a consent form to participate in the study and completed the survey within 24 hours of their first visit. We strictly used 24 hours rule to capture their initial experience fresh right after their very first visit with their cancer doctor. Due to COVID-19 restrictions, we have administered the survey over the phone. Each participant completing the survey was given a US $30 gift card. Data collection was conducted from February 2021 through May 2022. No participant identifiers were obtained during the study. Based on Green’s rule of thumb, for regression and correlation analysis, the sample size should be larger than 50 participants [47]. In our study, we aimed for 130 to 150 participants. By May 2022, we had received 135 participants. The participants were seen by 13 doctors. We limited the number of patients seen by each doctor to a maximum of 15 patients per doctor. We recruited patients with various cancer diagnoses. However, the majority of them were diagnosed with breast cancer, lymphoma, and multiple myeloma. We had 58 female participants (Table 1) and 45 participants from minority groups (Hispanic and African American). Most participants were between the ages of 50-64 years old.

**Table 1:** The demographics of the participants included in the study.

Demographics		Participants (N=135), n (%)
**Age (years)**		
	18-34	7 (5.19)
	35-49	35 (25.93)
	50-64	91 (67.41)
	>64	2 (1.48)
**Education**		
	No diploma	4 (2.96)
	Some school	17 (12.59)
	High school	44 (32.59)
	Technical college	20 (14.81)
	Bachelor	34 (25.19)
	Grad school or more	16 (11.85)
**Race**		
	Black American	28 (20.74)
	Hispanic	17 (12.59)
	White	81 (60)
	Other	9 (6.67)
**Gender**		
	Male	77 (57.04)
	Female	58 (42.96)

### Instrumentation

We developed our survey using validated instruments from the literature. The questions included in this survey measure the perceived workload, trust towards doctors, EHR use perception, and patient satisfaction with the care received. We also captured the participants’ demographics (education level, age, race, and gender).

The perceived workload is captured through the NASA TLX index. NASA’s TLX index is a popular construct in human factors science [48]. It was shown to be among the most reliable and valid questionnaires to measure workload in health care settings [49]. As shown in Table 2, the NASA TLX index has 6 main components physical demand, temporal demand, mental demand, effort, frustration, and performance. Trust is captured through the doctors’ trust scale, and the technology used is captured through the perception of the computer use scale. The exact questions used to capture each variable are detailed in Multimedia Appendix 1.

**Table 2:** Variables of the study.

Category and variable		Scale or questions used
**Workload**		NASA TLX^a^ index
	Physical demand	
	Temporal demand	
	Mental demand	
	Effort	
	Frustration	
	Performance	
**Quality of care**		
	Doctor’s trust	Trust scale
	Technology use perception	EHR^b^ use a perception scale
	Satisfaction with care	How satisfied were you with the overall visit?

^a^NASA TLX: National Aeronautics and Space Administration Task Load Index.

^b^EHR: electronic health record.

**Figure 2:**
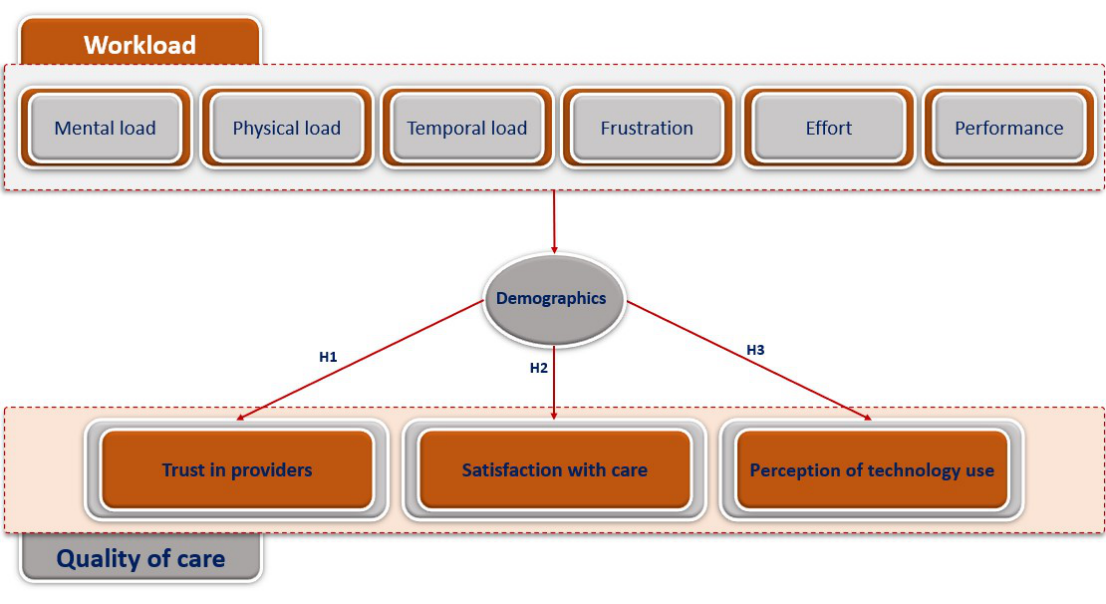
The detailed conceptual framework illustrates the hypotheses tested in this study. HIT: health information technology.

We adopted NASA TLX to capture workload experience by measuring mental, physical, temporal, performance, effort, and frustration components [50]. The NASA TLX has been validated for single-task environments [50,51]. The questions of the NASA scale compose an averaged 100 point-score. Originally, researchers applied a weighting procedure to the raw test scores of NASA TLX to develop a composite score tailored to individual workload definitions, however many researchers have eliminated the weighting procedure and instead use the raw test scores since it is simpler to apply: the ratings are averaged or added to create an estimate of overall workload between 0-100 [49]. In addition, we dichotomized the variables as follows: a value of 30 points and more is considered a high workload [52]. We also followed the same logic for the cut-off of high and low for specific components of NASA TLX. Trust in doctors is measured in this study using the subscale “trust in health care providers” of the “Multidimensional Trust in Health Care Systems Scale,” developed and validated by Egede and Ellis [53]. It is an averaged score composed of 10 questions with 4 Likert scale answers [53]. We dichotomized the trust scale in a way that a score above 50% was considered a high trust. Technology use perception is measured through the averaged scale of “Patient-Reported Satisfaction with Physician Computer Use,” assessed and validated for electronic medical records and other computer uses in health care settings to evaluate patients’ perception of doctors’ use of computer systems [54]. For satisfaction with care, we use a 5-Likert scale question where patients are asked about their satisfaction with the visit. Both satisfaction with care and technology perception scales are dichotomized in a way that a score above 50% is considered high. We test the overall score and the components’ associations for each variable. Figure 2 shows the detailed conceptual framework of the study.

### The Nature of the First Cancer Visit and Tasks

It is essential to understand the nature of the visit and tasks in the first cancer visit to envision the workload for the patients. The first consultation with new cancer patients is spent on the following tasks:

1. Reviewing diagnosis of cancer, type of cancer, extent of cancer
2. Reviewing imaging studies performed and discussing any additional work-up that might be recommended (eg, breast magnetic resonance imaging, additional biopsies, other imaging studies)
3. Discuss treatment options (surgery, radiation, systemic treatment, plastic surgery), assuming most of the work-up is completed.
4. Assess general health status or other medical issues
5. Assess social support or mental health or coping
6. Assess for any clinical trials

The primary task for patients is engagement during these tasks. Some of these tasks are done by shared decision-making, so patients are required to understand discussed topics for their best interests.

### Statistical Analysis

First, we ran descriptive statistics for all the study variables. Second, logistic regression analysis was run for the scores and the components to explore the correlation between all the variables and test the hypothesis as shown in the framework (Figure 2). All the regression models were adjusted for the demographics (age, race, gender, and education level). Model variables were dichotomized for analysis purposes based on the information existing in the literature [55]. Confirmatory factor analysis (CFA) was performed using the survey measures to analyze the psychometric properties of the variables. The fit and reliability of the CFA to the data were determined as acceptable as indicated by commonly used metrics such as composite reliability greater than 0.90 [56], average variance extracted greater than 0.50 [57], Guttman lambda 6, and coefficient omega (for second-order CFA of expectancy) greater than 0.80 [58]. All data cleaning and analyses were done using Python 3.7 using some packages (eg, pandas, stats, numpy).

## Results

### Descriptive Analysis

**Figure 3:**
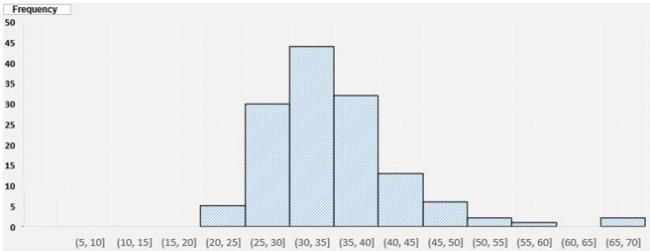
National Aeronautics and Space Administration Task Load Index (NASA TLX) composite range based on the number of participants.

Figure 3 shows the distribution of the overall workload across the participants. The lowest workload we observed was around 20-25 out of 100 (7/135, around 5% of the participants), whereas the highest level of workload was around 65-70 out of 100. Overall, the majority of patients reported a high workload (score >30).

Table 3 shows the percentages of participants who have low and high workloads across different demographics. As shown in Table 3, (108/135, 80%) of patients experienced an overall high level of workload based on NASA TLX scores. However, when we look at the specific components, we see that 55% (75/135) of the patients experienced a high mental load, which is the question of mental activity to perform activities such as thinking, deciding, remembering, etc. On the other hand, we also see that participants reported a low level of effort (71.1%, 96/135); time pressure felt due to the rate or pace at which tasks occurred during the visit (88.89%, 120/135); low frustration due to feeling insecure, discouraged, irritated, stressed, and annoyed (115/135, 85.2%); and the perception that low levels of physical activity were required from them to perform activities in the visit (94/135, 69.93%).

**Table 3:** The distribution of the workload trends among the different demographic subgroups.

Demographics			NASA TLX^a^ score, n (%)				Frustration, n (%)				Performance, n (%)				Effort, n (%)				Time load, n (%)				Physical load, n (%)				Mental load, n (%)	
			Low		High		Low		High		Low		High		Low		High		Low		High		Low		High		Low	High
**Age (years)**																												
	18-34 (n=7)	0 (0)		7 (100)		7 (100)		0 (0)		0 (0)		7 (100)		5 (71.43)		2 (28.57)		6 (85.71)		1 (14.29)		5 (71.43)		2 (28.57)		4 (57.14)		3 (42.86)
	35-49 (n=35)	9 (25.71)		26 (74.29)		31 (88.57)		4 (11.43)		0 (0)		35 (100)		25 (71.43)		10 (28.57)		33 (94.29)		2 (5.71)		27 (77.14)		8 (22.86)		21 (60)		14 (40)
	50-64 (n=91)	18 (19.78)		73 (80.22)		74 (82.42)		17 (17.58)		2 (2.20)		89 (97.80)		66 (72.53)		25 (27.47)		81 (89.01)		10 (10.99)		62 (68.13)		29 (31.87)		37 (40.66)		54 (59.34)
	>64 (n=2)	0 (0)		2 (100)		2 (100)		0 (0)		0 (0)		2 (100)		0 (0)		2 (100)		0 (0)		2 (100)		0 (0)		2 (100)		1 (50)		1 (50)
**Education**																												
	No diploma (n=4)	0 (0)		4 (100)		3 (75)		1 (25)		0 (0)		4 (100)		3 (75)		1 (25)		4 (100)		0 (0)		2 (50)		2 (50)		1 (25)		3 (75)
	Some school (n=17)	1 (5.88)		16 (94.12)		16 (94.12)		1 (5.88)		0 (0)		17 (100)		14 (82.35)		3 (17.65)		16 (94.12)		1 (5.88)		14 (82.35)		3 (17.65)		9 (52.94)		8 (47.06)
	High school (n=44)	9 (20.45)		35 (79.55)		37 (84.09)		7 (15.91)		1 (2.27)		43 (97.73)		33 (75)		11 (25)		40 (90.91)		4 (9.09)		29 (65.91)		15 (34.09)		21 (47.73)		23 (52.27)
	Technical college (n=20)	7 (35)		13 (65)		17 (85)		3 (15)		1 (5)		19 (95)		16 (80)		4 (20)		17 (85)		3 (15)		15 (75)		5 (25)		11 (55)		9 (45)
	Bachelor (n=34)	7 (20.59)		27 (79.41)		29 (85.29)		5 (14.71)		0 (0)		34 (100)		20 (58.82)		14 (41.18)		28 (82.35)		6 (17.65)		24 (70.59)		10 (29.41)		15 (44.12)		19 (55.88)
	Grad school or more (n=16)	3 (18.75)		13 (81.25)		13 (81.25)		3 (18.75)		0 (0)		16 (100)		10 (62.50)		6 (37.50)		15 (93.75)		1 (6.25)		10 (62.50)		6 (37.50)		6 (37.50)		10 (62.50)
**Race**																												
	Black American (n=28)	6 (21.42)		22 (78.57)		26 (92.59)		2 (7.41)		1 (3.70)		27 (96.30)		22 (78.57)		6 (21.42)		25 (88.89)		3 (11.11)		21 (74.07)		7 (25.93)		17 (59.26)		11 (40.74)
	Hispanic (n=17)	4 (23.53)		13 (76.47)		14 (88.24)		3 (11.76)		0 (0)		17 (100)		11 (64.71)		6 (35.29)		16 (94.12)		1 (5.88)		13 (76.47)		4 (23.53)		7 (41.18)		10 (58.82)
	White (n=81)	14 (17.95)		67 (82.05)		68 (83.33)		13 (16.67)		1 (1.28)		80 (98.72)		58 (71.79)		23 (28.21)		72 (88.89)		9 (11.11)		52 (64.10)		29 (35.90)		35 (43.21)		46 (56.79)
	Other (n=9)	2 (22.22)		7 (77.78)		6 (66.67)		3 (33.33)		0 (0)		9 (100)		6 (66.67)		3 (33.33)		7 (77.78)		2 (22.22)		8 (88.89)		1 (11.11)		6 (66.67)		3 (33.33)
**Gender**																												
	Male (n=77)	19 (24.68)		58 (75.32)		66 (85.71)		11 (14.29)		0 (0)		77 (100)		51 (66.23)		26 (33.77)		66 (85.71)		11 (14.29)		54 (70.13)		23 (29.87)		43 (55.84)		34 (44.16)
	Female (n=58)	8 (13.79)		50 (86.21)		49 (84.48)		9 (15.52)		2 (3.45)		56 (96.55)		45 (77.59)		13 (22.41)		54 (93.10)		4 (6.90)		40 (68.97)		18 (31.03)		19 (34.48)		39 (65.52)
All (N=135)			27 (20)		108 (80)		115 (85.19)		20 (14.81)		2 (1.48)		133 (98.52)		96 (71.11)		39 (28.89)		120 (88.89)		15 (11.11)		94 (69.63)		41 (30.37)		63 (46.67)	72 (53.33)

^a^NASA TLX: National Aeronautics and Space Administration Task Load Index.

### Impact of Workload on Quality of Care

**Figure 4:**
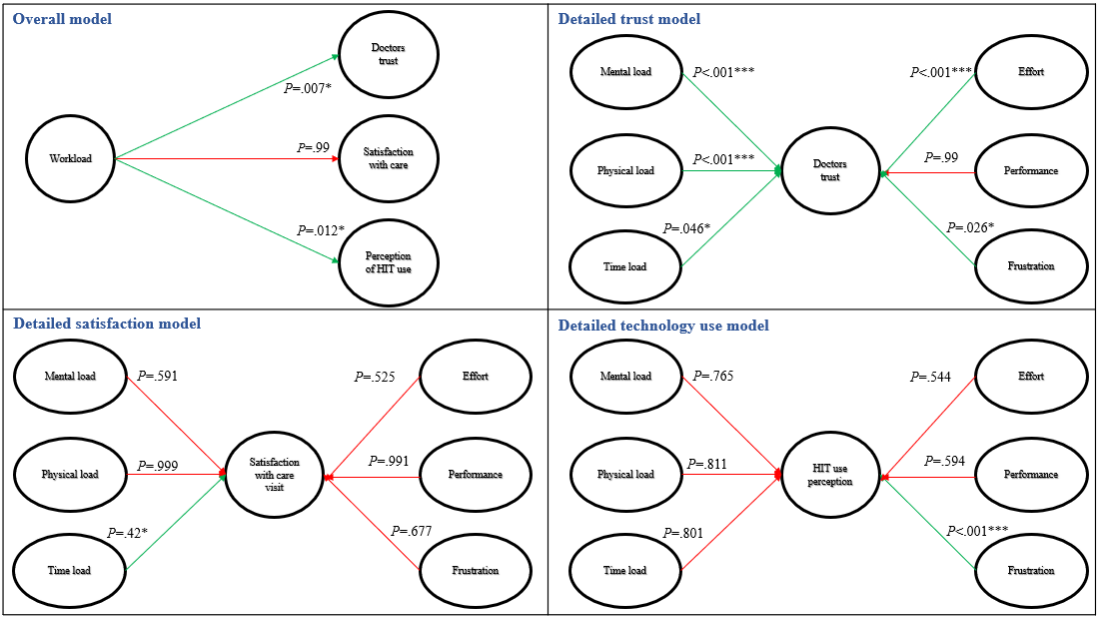
Detailed results for the conceptual model’s validation. HIT: health information technology. *P<.05; *P*<.01; *P*<.001.

Figure 4 shows the results of the different models we tested. The first model shows the relationship between the overall NASA TLX score and its relationship with 3 outcomes. The other 3 models show the relationship between each component of NASA TLX (mental load, physical load, time load, effort, performance, and frustration) and outcome measures (trust, satisfaction, and perception of technology use). As shown in Figure 4, the more overall workload patients felt, the less they trusted their doctors (odds ratio [OR] 0.059, 95% CI 0.001-2.34; *P*=.007). We, thus, fail to reject hypothesis 1. Low trust was also associated with the demanding mental tasks in the visits (OR 0.055, 95% CI 0.002-2.64; *P*<.001), the physical load (OR 0.194, 95% CI 0.004-4.23; *P*<.001), the time load (OR 0.183, 95% CI 0.02-2.35; *P*=.046), the effort needed to cope with the environment (OR 0.163, 95% CI 0.05-1.69; *P*<.001), and the frustration levels (OR 0.323, 95% CI 0.04-2.55; *P*=.03). Patient’s performance during the visits did not impact their trust in their doctors.

When we look at the model for satisfaction, the overall workload level did not impact the patients’ satisfaction with the overall visit. We, thus, reject hypothesis 2. The detailed satisfaction model also showed that only 1 specific component significantly impacts care satisfaction. The patient’s satisfaction with the overall visit was negatively impacted by the time load they experienced (OR 0.123, 95% CI 0.001-2.56; *P*=.04), as shown in Figure 4.

Finally, patients’ perceptions of EHR use during the visit were negatively impacted by the overall workload experienced by the patients (OR 0.315, 95% CI 0.08-6.35; *P*=.01) and the high frustration level experienced (OR 0.111, 95% CI 0.015-3.75; *P*<.001), as shown in Figure 4. We, thus, fail to reject hypothesis 3.

## Discussion

### Principal Results

Doctor workload has been studied by several studies in the human factors field. However, there is a clear gap in the literature investigating the workload of patients during visits, especially in a complex environment such as cancer. This is the first study to explore cancer patients’ workload and its associations with various outcomes (doctors’ trust, use of technology perception, satisfaction with care) during the visit.

### Summary of Findings

Encounters in cancer care might be stressful and cognitively highly demanding for patients and doctors. Studies have already shown that doctors have moderate to high workloads, even in primary care settings [38]. In our study, we also observed that most of the participants (108/135, 80%) experienced a somewhat high workload during the visit across various demographics. The various models we tested yielded interesting results. The overall NASA TLX workload scores had a significant association with the patient’s trust in doctors as well as the patient’s perception of technology use (doctor’s EHR use) during the visit. However, we did not observe a significant association with the satisfaction score.

According to our findings, the high workload perceived by patients during the visit results in less trust in their doctors. The detailed components of NASA TLX, including patients’ frustration in addition to the effort, mental, physical, and time load required to perform activities during the visits, also impacted the patients’ trust in their doctors. This interesting finding has implications for reconsidering and redesigning the structure of the first visits. Building trust and rapport between doctor and patient on the first visit is critical and requires high-quality communication skills [59]. In addition, many factors were shown to impact trust in the literature due to its fragility, such as the rapid changes in the health care system and conditions of care [60]. In response to the cancer diagnosis, patients experience emotional and physical impairment coupled with developing a sense of transitoriness (finitude of life) [61]. It becomes hard for them to adapt to the new situation and find continuity in their lives in the middle of the flow of information and decisions they should deal with [61]. This may explain the association between the high workload and low trust noticed among new cancer patients. A study by Plomp and Ballast [62] investigating the vulnerability of doctor-patient trust in occupational health showed that in critical sensitive situations, a high workload creates a vulnerability in patients, resulting in more difficulty trusting their doctors. The authors state that “a combination of poor health and high workload could create a greater (need to) trust but is obviously not a sufficient condition to overcome stubborn distrust” [62].

We also noted a significant relationship between workload and patients’ perception of the use of technology during the visits. In fact, new cancer patients experience a range of emotions, including shock, sadness, anger, disappointment, and confusion [63]. The added anxiety of not knowing the next steps can cause even more stress and frustration [63]. The emotional burden was found to be highly associated with their perception of the quality of care and life among newly diagnosed lung cancer patients [63]. As new cancer patients would still be building their communication paths with their doctors, technology use during the visit might add to the high workload and improve the frustration of the patients during these emotional visits. This also might indicate that patients may not prefer technology used within the visits to be able to spend more time with their doctors and feel well listened to. In addition, the detailed model also yielded an interesting result showing only 1 component of NASA TLX: the frustration variable concerning if the patients felt annoyed, stressed, or discouraged, which has a significant association with perceived technology use. This is an interesting result supporting some of the early studies done in primary care. Despite the potential role of technology in strengthening the therapeutic alliance between doctors and their patients [46], researchers have argued that using computers during visits, especially under emotional situations, may negatively impact interaction as it does not allow the patients to find their way of decoding nonverbal information appropriately and may prevent them from building cue channels of interactions with their doctors [64].

Finally, the high level of workload did not impact patient satisfaction with the visits. Only the time load negatively impacts satisfaction. This also shows that time pressure during cancer visits might influence satisfaction negatively. Given that this is their first visit as cancer patients, they want to use all necessary time to discuss their concerns and do not want to feel rushed during the visit. Some studies also argued that cancer patients’ satisfaction with care is associated with the timeliness of care, as cancer patients have a load that exceeds the time available to them [65]. In addition to the increased susceptibility to stress resulting from the diagnosis, the patient’s anxiety can be amplified by long waiting times for appointments and results and long medical visits, which negatively impacts the patients’ satisfaction with the quality of care delivered [66].

Even though the NASA TLX index was designed specifically for aviation occupations, it has proved its use in different industries [67,68]. In health care, it was shown to be effective in measuring doctors’ workload in various critical environments to explore the impact of technology use on their activities [69]. In a study by Lund et al [70], it was used to measure the workload levels of surgeons to evaluate the association between their burnout and their performance. It showed high levels of workload after long working shifts. It was used by Norasi et al [71] to evaluate the usability of the robots to support the surgeons’ workload and teamwork effectiveness. It was also used to test the effectiveness of using augmented reality technologies to support cognitive demand [72]. Thus, in addition to its role in evaluating the usability of technology in health care, we showed that the NASA TLX index has the potential to support researchers in evaluating the workload of patients in cancer care.

### Practical Implications

Theoretically, it is feasible to presume that newly diagnosed cancer patients experience a high workload. However, in practice, it remains important to investigate the impact of the high workload on patients’ quality of care perception to suggest corrective strategies based on the patients’ needs and performance. Our findings also have theoretical implications. First, most of the studies investigating workload in health care explore it from a doctor’s perspective accounting for their performance boosters to create a good work environment. Our study is the first study in the field of human factors that investigates workload among patients and captures its direct impact on their perception of care quality (trust in doctors, satisfaction with care, perception of technology use). Identifying the direct factors impacted by workload adds to the literature on the predictors of the quality of cancer care. Learning what influences the overall rating of care can enable doctors to accommodate vulnerable patient groups. Identifying health care aspects that are independently associated with the overall rating of care may enable targeted efforts when planning and prioritizing initiatives to improve the patient-experienced quality of care. Furthermore, as technology use was associated with a high workload in our analysis, more thought should be given to better design simplification and better system integration to control the physical and cognitive workload among patients as well as doctors. The clear impactful interactions between doctors, patients, and technology raise a flag for the importance of considering this trio in the different interventions made in cancer care to make sure to involve all parts of the equation. This will make “patient work” less demanding and more accurate, which includes understanding the situation and making the right shared decision in the cancer treatment during the first cancer visit.

### Limitations and Future Studies

This study has some limitations that should be acknowledged. First, the study is cross-sectional and captures the patients’ opinions at a certain point in time. Future studies should involve longitudinal data and explore the proposed relationships over time to compare the same findings throughout different stages of cancer (treatment vs diagnosis) and observe the evolution. Second, patients participated in the study at a very early stage after diagnosis. Despite the originality of the findings, this may add more bias to their perception of their workload. A follow-up after some days should be done to validate their perceptions. Some environmental factors, like the crisis related to COVID-19, may add more pressure to the patient’s situation, which may bias the results related to the emotional load and the frustration level. Better control of environmental factors would increase the validity of the data from various measurements. Apart from addressing our limitations, there is room for additional future research based on our findings. Future research also should explore the workload of doctor and patient dyads who are on the same visit to compare the workload assessment and factors leading to workload in both parties. Researchers should also test various technology designs and explore how their use might improve the perceived workload of both doctors and patients during the visits.

### Conclusions

We showed that most patients with cancer in the study experienced a high workload based on NASA TLX scores. The overall workload is also associated significantly with patient trust in the doctor as well as the perception of EHR use during the visit, but it does not impact satisfaction significantly. Future human factors work might explore the workload and driving factors in longitudinal studies and assess whether these workloads might contribute to unintended patient outcomes and medical errors. Finally, better technology design and use can minimize perceived workload, which might contribute to the trust relationship between doctors and patients in this critical environment.

## Appendix

Multimedia Appendix 1 Survey questions.

## Abbreviations

CFA: confirmatory factor analysis
EHR: electronic health record
NASA TLX: National Aeronautics and Space Administration Task Load Index
OR: odds ratio
