# Supplementary material for: Perceived Patient Workload and Its Impact on Outcomes During New Cancer Patient Visits: Analysis of a Convenience Sample
Source: JMIR Hum Factors. 2023 Aug 18;10:e49490. doi: 10.2196/49490 (PMC10474510; doi:10.2196/49490)
Supplement: Multimedia Appendix 1 [file humanfactors_v10i1e49490_app1.docx]

Multimedia Appendix 1

| Scale | Questions | Answers’ scale |
| --- | --- | --- |
| NASA-TLX index | - (Mental demand). How much mental activity was required to perform activities in your visit (thinking, deciding, calculating, remembering, looking, etc.)? - (Physical demand). How much physical activity was required to perform activities in the visit related to interaction? - (Temporal demand). How much time pressure did you feel due to the rate or pace at which the tasks or task elements occurred in the visit? - (Performance) How successful were you in accomplishing what you were asked to do? How satisfied were you with your performance/interaction at the visit? - (Effort) How hard did you have to work/think to accomplish a good outcome from the visit? - (Frustration) How insecure, discouraged, irritated, stressed, and annoyed were you? | 1 (very low)  2  3  4  5  6  7  8  9  10 (very high) |
| Trust scale | - My health care provider was considerate of my needs and put them first - I have so much trust in my health care provider that I will always try to follow his/her advice - I trust my health care provider so much that whatever he/she tells me, it must be true - I trust my health care provider’s opinion, and therefore I feel I don’t need a second one - I can trust my health care providers’ judgments concerning my medical/cancer care - My health care provider would do whatever it takes to give me the medical care that I need - Because my health care provider is an expert, he was able to treat medical problems like mine - I can trust my health care provider’s decisions on which medical treatments are best for me - My health care provider offered me the highest quality of medical care - All things considered; I completely trust my health care provider | 1) not at all  2) not very much  3) somewhat  4) a lot  5) a great deal |
| EHR use perception | - The doctor’s computer use helped me better understand what happened today. - The computer helped the provider know about all the things happening in my medical care. - The computer helped the provider make my care more personalized. - The computer use helped the visit run in a timelier manner. - I felt frustrated when the doctor used the computer. - I wanted to know what the doctor was typing into the computer. - The doctor’s computer use was distracting. - I felt comfortable speaking to the doctor while he/she was typing. - The doctor relied heavily on the computer. - My provider listened to me less care because of the computer in the room - My provider looked less at me because of the computer in the room - Computer use interfered with my communication with the doctor. - Computer use made the visit feel less personal. - Overall, I liked the way that the provider used the computer in today’s visit | 1) Strongly Disagree  2) Disagree  3) Neither  4) Agree  5) Strongly Agree |
| Satisfaction with care | - - How satisfied were you with the overall visit? | 1) not at all  2) not very much  3) somewhat  4) a lot  5) a great deal |
